# Clinical challenges of an Xp21 contiguous gene deletion syndrome in a newborn and 15 months of follow-up - case report

**DOI:** 10.3389/fendo.2026.1858779

**Published:** 2026-07-20

**Authors:** Ignacy Frulenko, Iwona Ostrowska, Michał Patalan, Aida Bertoli-Avella, Nayla Y. León, Andreia Pinto, Peter Bauer, Alicja Leśniak, Daria Katuszonek, Marta Glińska, Monika Modrzejewska, Robert Śmigiel, Maria Giżewska

**Affiliations:** 1 Faculty of Medicine, Pomeranian Medical University in Szczecin, Szczecin, Poland; 2 Department of Pediatrics, Rare Diseases and Metabolic Medicine, Pomeranian Medical University in Szczecin, Szczecin, Poland; 3 Centogene GmbH, Rostock, Germany; 4 Pomeranian Medical University in Szczecin, Szczecin, Poland; 5 Department of Pediatrics, Endocrinology, Diabetology, Metabolic Diseases and Cardiology of the Developmental Age, Prof. Tadeusz Sokołowski University Teaching Hospital No. 1, Szczecin, Poland; 6 2nd Department of Ophthalmology, Pomeranian Medical University in Szczecin, Szczecin, Poland; 7 Department of Pediatrics, Endocrinology, Diabetology and Metabolic Diseases, Wrocław Medical University, Wrocław, Poland

**Keywords:** case report, contiguous gene deletion syndrome, Duchenne muscular dystrophy, glycerol kinase deficiency, neurodevelopmental delay, primary adrenal insufficiency, Xp21 deletion syndrome

## Abstract

Xp21 contiguous gene deletion syndrome is a rare X-linked disorder caused by interstitial deletions involving neighboring genes such as *NR0B1*, *GK*, *DMD*, and *IL1RAPL1*, and is clinically associated with primary adrenal insufficiency, glycerol kinase deficiency, Duchenne muscular dystrophy, and neurodevelopmental delay. The phenotype depends on deletion size and gene content. We report a male child with a 4.5 Mb hemizygous deletion at Xp21.3-p21.2 encompassing *IL1RAPL1*, *NR0B1*, *GK*, and exons 61 to 79 of *DMD*, confirmed by exome sequencing and shown to be maternally inherited. The presented patient was admitted at 8^th^ day of life due to concerns for primary muscular or metabolic disease. During the second week of life, the patient developed salt-wasting primary adrenal insufficiency with hyponatremia and hyperkalemia. Persistently elevated creatine kinase exceeding 3000 U/L and markedly elevated cardiac biomarkers were observed in the absence of structural and functional cardiac abnormalities. Urinary organic acid analysis demonstrated significant glyceroluria, and severe serum hypertriglyceridemia was attributed to pseudo-hypertriglyceridemia consistent with glycerol kinase deficiency. Early visual impairment with nystagmus and pale optic discs were noted, and by 15 months the child exhibited global developmental delay, most pronounced in gross motor and language domains. This case illustrates the diagnostic challenges and clinical complexity of Xp21 contiguous gene deletion syndromes. In patients with Xp21 deletion syndrome, early molecular diagnosis enables timely hormone replacement, metabolic surveillance, cardiologic follow-up, and structured neurodevelopmental care.

## 1 Introduction

The Xp21 contiguous gene deletion syndrome (OMIM: 300679, ORPHA: 261476), previously known as complex glycerol kinase deficiency, encompasses the deletion of several genes presenting as a blended phenotype of primary adrenal insufficiency, Duchenne muscular dystrophy, hyperglycerolemia and neurodevelopmental delay. The unifying diagnosis of Xp21 deletion syndrome includes disease entities, which may exist as separate disorders and have a differing clinical presentation. The clinical features depend on the deletion size and the number and type of genes involved. The impact of multi-gene deletions on early metabolic decompensation, cardiac biomarkers, and neurodevelopment is incompletely understood. Here, we report a patient with Xp21.3–p21.2 deletion syndrome involving four clinically relevant genes: *IL1RAPL1*, *NR0B1*, *GK*, and partially *DMD*, who presented with early-onset adrenal insufficiency, metabolic disturbances, and visual impairment. This case highlights the diagnostic challenges and clinical complexity of Xp21 contiguous gene deletion syndrome and contributes to the expanding genotype-phenotype correlations associated with this rare condition.

## 2 Case presentation

**Figure 1:**
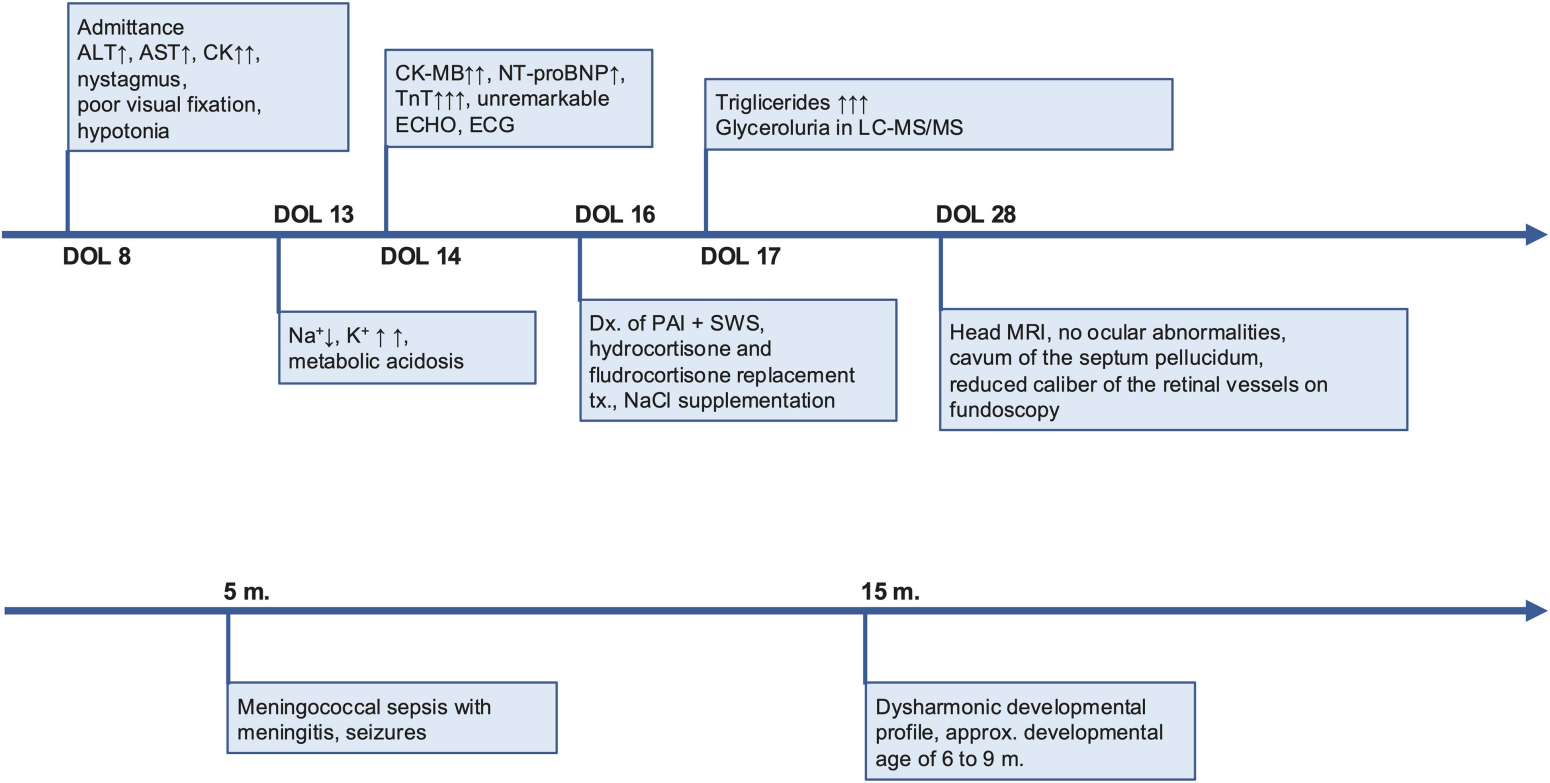
Timeline of the events described in the case report. DOL, Day of Life; ALT/AST, Alanine/Aspartate Aminotransferase; CK, Creatine Kinase; CK-MB, Creatine Kinase, Myocardial Band; NT-proBNP, N-terminal pro-B-type Natriuretic Peptide; TnT, Troponin T; ECHO, Echocardiography; ECG, Electrocardiogram; LC-MS/MS, Liquid Chromatography, Tandem Mass Spectrometry; dx., Diagnosis; PAI, Primary Adrenal Insufficiency; SWS, Salt-Wasting Syndrome; tx., Treatment; MRI, Magnetic Resonance Imaging; m., Months of age.

A full-term Caucasian male newborn (gestational age 39 weeks), delivered by cesarean section, from the first pregnancy (Apgar score 10/10, birth weight 3590 g), was transferred to the Department of Pediatrics on the 8^th^ day of life (DOL), with a suspicion of hepatic or muscular injury (for event timeline see **Figure 1**). During pregnancy, his 24-year-old mother, previously diagnosed with mild intellectual disability, experienced a seizure and was diagnosed with epilepsy. Lamotrigine was initiated as antiepileptic therapy and was continued from the third trimester onward. The patient’s first days of life were complicated by intermittent respiratory distress, he required incubator care, respiratory support and presented decreased muscle tone. On admission, at DOL 8, the infant presented with jaundice and mild muscular hypotonia. A detailed physical examination revealed no additional abnormalities, except for poor visual fixation and subtle nystagmus. Over the following days, the initially concerning laboratory parameters decreased but remained significantly above the upper reference limits, with creatine kinase (CK) levels reaching around 3000 U/L (reference range (ref.): 70–380 U/L). Abdominal, cranial, and lung ultrasonography (US), as well as echocardiography (ECHO) and electrocardiography (ECG), revealed no significant abnormalities. Additional tests, including coagulation parameters, ammonia, albumin, renal function tests, and blood glucose levels, were within normal limits. Beginning on DOL 13 he developed initially asymptomatic hyponatremia (lowest serum sodium concentration: 123 mmol/L; ref. 135–145 mmol/L), which was resistant to both oral and intravenous sodium chloride supplementation. This was accompanied by mild metabolic acidosis and hyperkalemia. On DOL 14, a further increase in CK-MB, NT-proBNP, and troponin T concentrations was observed, while total CK values remained above 3000 U/L (see **Table 1**). Repeat cardiac studies were unremarkable. TORCH infections were excluded by negative serology. Biliary atresia was also unlikely, due to the falling total and direct bilirubin levels and gradual resolution of the jaundice. Initially, toxic liver injury or myocarditis related to *in-utero* lamotrigine exposure was suspected, however, this was ruled out because lamotrigine was undetectable in the infant’s blood. Persistent hypotonia and sustained elevation of CK levels raised suspicion of an underlying congenital muscle disorder. As part of the diagnostic workup for inherited metabolic disorders (IMD), blood acylcarnitine profiling and urinary organic acid analysis were performed using tandem mass spectrometry (LC-MS/MS) and gas chromatography–mass spectrometry (GC-MS), respectively.

**Table 1:** Select serial laboratory parameters by day of life (DOL).

Parameter	DOL 9	DOL 11	DOL 12	DOL 14	DOL 15	DOL 20	DOL 26	DOL 32	DOL 33
Sodium, Na (mmol/L) ref. 132 – 147	130	130		130	130	128	124	134	
Potassium, K (mmol/L) ref. 3.6 – 6.1	7.75	5.96		8.01	6.96	7.6	6.88	5.84	
AST (U/L) ref. <50	146			102		70			
ALT (U/L) ref. <50	84			62		59			
GGTP (U/L) ref. 9 - 60	691	623		541		430	258		
Bilirubin, total (mg/dL) ref. 0.3 – 1.1	10.9	8.17				1.99			
Bilirubin, direct (mg/dL) ref. <0.3	1.09	0.71				0.86			
Troponin T (ng/L) ref. >100 ng/L – high risk of myocardial injury		440	672	911	759	379	274		
CK (U/L) ref. <252	8928		3855	3906	3033	1606	3338		
CK-MB (U/L) ref. 7 – 25		156	203	221	180	148	178		
NT-proBNP (pg/mL) ref. <125			460	516			214		
pH ref. 7.35 – 7.45	7.413			7.398	7.391	7.36	7.433		7.419
pCO2 (mmHg) ref. 35 - 45	33.2			30.6	32	33.6	34.4		39.4
pO2 (mmHg) ref. 80 – 100	65.6			62.6	64.4	55.4	70.5		57.3
HCO3 (mmol/L) ref. 21 – 26	20.7			18.4	19	18.6	23.5		24.9
tCO2 (mmol/L) ref. 22 -26	19.4			19.4	20	19.6	NA		26.1
Base excess (mmol/L) ref. - 3 – 3	-2.9			-5.1	-4.8	-5.8	-1.1		0.5
ACTH (pg/mL) ref. 7.2 – 63.3					207	536	32		

AST, aspartate aminotransferase; ALT, alanine aminotransferase; GGTP, γ-glutamyl transpeptidase; CK, creatine kinase; CK-MB, creatine kinase MB fraction; NT-proBNP, N-terminal pro-B-type natriuretic peptide; HCO3, bicarbonate; tCO2, total carbon dioxide; ACTH, adrenocorticotropic hormone; NA, not available (sample analyzed on a Cobas b 221 analyzer, which does not report tCO2). Blank cells indicate the parameter was not measured on that day.

Since admission, the patient experienced intermittent diarrhea and regurgitation, accompanied by slow weight gain. Serum cortisol concentrations remained within the reference range, whereas adrenocorticotropic hormone (ACTH) levels were elevated. In addition, very high renin concentrations (>500.0 µIU/mL; ref. 2.8–39.9 µIU/mL) were observed, accompanied by relatively low aldosterone levels. Progressive elevation of ACTH concentration (up to 536.0 pg/mL) together with insufficient cortisol secretion (16 µg/dL) during the short Synacthen (tetracosactide) stimulation test established the diagnosis of primary adrenal insufficiency (PAI) with salt-wasting syndrome (SWS). The patient had not received glucocorticoid therapy prior to the test. Congenital adrenal hyperplasia (CAH) was excluded based on low 17-hydroxyprogesterone (17-OHP) in newborn screening, confirmed by repeated measurements. Hydrocortisone replacement therapy was initiated intravenously and later transitioned to oral administration, together with fludrocortisone and oral sodium supplementation. Gradual normalization of biochemical parameters (serum sodium and potassium, acid–base status, ACTH, and GGTP), along with improved weight gain was observed thereafter. The patient did not develop hyperpigmentation at any time point while under care. Repeat abdominal US demonstrated that the adrenal glands were not clearly discernible.

**Figure 2:**
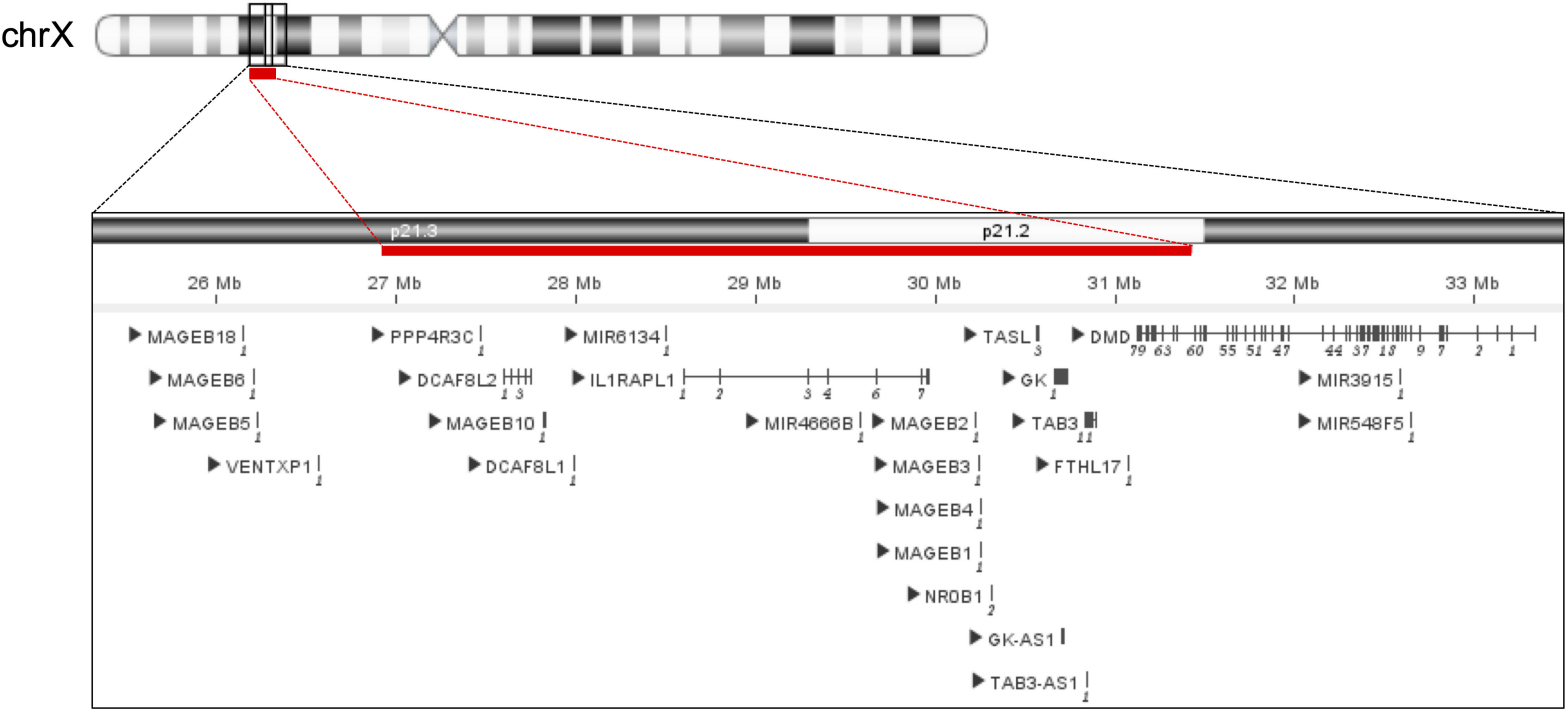
A visual representation of the deleted region found in the index, red band directly indicates the deletion. The deleted *DMD* exons (61 to 79) are a source of transcript variants corresponding to dystrophin isoforms Dp71 (NM_004015.3), Dp71b (NM_004016.3), Dp71a (NM_004017.3), Dp71ab (NM_004018.3) and Dp40 (NM_004019.3) which are implicated in the pathogenesis of cognitive and visual deficits in DMD patients.

Further laboratory testing revealed marked hypertriglyceridemia (1149 mg/dL; ref. ≤150 mg/dL), and urinary organic acid analysis by GC-MS demonstrated significant glyceroluria. Glycerol kinase deficiency (GKD) was therefore suspected, and the elevated triglyceride concentrations were interpreted as pseudo-hypertriglyceridemia. The combination of significant glyceroluria, PAI, and hypotonia with persistent hyperCKemia suggested a contiguous gene deletion syndrome involving Xp21. To confirm the diagnosis, exome sequencing (ES) analysis was performed (Centogene GmbH, Rostock, Germany). A pathogenic interstitial hemizygous deletion of approximately 4495 kb was identified within the Xp21.3–Xp21.2 chromosomal region (see **Figure 2**; **Table 2**), affecting four genes with established clinical relevance: *DMD, GK, IL1RAPL1*, and *NR0B1* (seq[GRCh37] Xp21.3p21.2(26936592_31431584)x0). Thus, the suspected diagnosis of Xp21 deletion syndrome was molecularly confirmed. Chromosomal microarray analysis (CMA) performed in the mother revealed the same deletion in a heterozygous state (arr[GRCh37] Xp21.3p21.2(26936592_31431584)x1), confirming the copy number variation (CNV) found in the index was maternally inherited. Given the mother’s diagnosis of mild intellectual disability and epilepsy, ES analysis was also performed for the mother, but no other clinically relevant variant was detected.

At 4 weeks of age, due to nystagmus and disturbances in visual fixation, cranial magnetic resonance imaging (MRI) was performed. The MRI revealed a 3.5-mm-wide cavum of the septum pellucidum; however, neither the optic nerves nor the structure of the visual pathways showed abnormalities. Fundoscopic examination demonstrated sharply demarcated, pale optic nerve discs at the level of the retina, and a significantly reduced caliber of the retinal vessels, in particular arteries.

At 5 months of age, the child was admitted to the emergency department with vomiting, fever, and severe distress, starting 4 hours prior to admission. At home, he received antipyretics and a triple dose of oral hydrocortisone. On admission he presented with signs of shock and petechiae on the trunk, extremities, and face. Management included intravenous fluid resuscitation, stress-dose hydrocortisone, and empiric ceftriaxone. Cerebrospinal fluid analysis revealed pleocytosis (9823 cells/µL, 87.2% polymorphonuclear leukocytes) elevated protein levels, and PCR confirmed Neisseria meningitidis. The course was complicated by transient hyponatremia and myoclonic seizures, treated with hypertonic saline, fluid restriction, and clobazam. Transfontanelle US was normal.

At 15 months of age, the child presented with a disharmonic developmental profile. Gross motor development was delayed, with preserved head control, the ability to assume a high prone position supported on extended upper limbs, and the ability to roll from supine to prone and vice versa. Independent sitting had not been achieved. Fine motor skills were developed relatively better. According to developmental milestones and standardized developmental assessment scores (e.g., the Bayley Scales of Infant and Toddler Development), the motor and language abilities corresponded to an approximate developmental age of 6–9 months. Language development was limited to single-syllable vocalizations. Visual tracking and an appropriate social smile were present.

Currently, the patient remains under systematic multidisciplinary follow-up and is undergoing intensive rehabilitation. He receives oral hydrocortisone (15–17 mg/m²/day) and fludrocortisone replacement therapy, with doses gradually reduced with age from 0.2 mg/day to 0.05 mg/day under renin and electrolyte monitoring, along with continued oral sodium supplementation.

## 3 Discussion

Chromosome Xp21 deletion syndrome is a rare contiguous gene deletion disorder, classically characterized by complex glycerol kinase deficiency, congenital adrenal hypoplasia, intellectual disability, and/or dystrophinopathy, predominantly affecting males. The clinical phenotype is variable and largely dependent on the size of the deletion and the specific genes involved. Developmental delay has been consistently reported in males with Xp21 deletions involving *DMD*, particularly when the deletion also includes *IL1RAPL1* (1, 2). To date, at least four additional male patients have been reported with deletions encompassing the same four clinically relevant genes involved in the present case (*DMD, GK, NR0B1*, and *IL1RAPL1*), with deletion sizes estimated at approximately 3–4 Mb and overlapping genomic coordinates. In these cases, clinical onset was early, typically within the neonatal period to the first few months of life (3–5). Hypotonia was a consistent finding across all cases, including our patient, while PAI, psychomotor delay, strabismus, and failure to thrive were commonly observed. One reported patient showed a predominantly muscular presentation with a later onset at 7 months (5), suggesting some phenotypic variability even among similarly sized deletions. Notably, among the five documented cases (including the current report), maternal genetic analysis was available in only two, both demonstrating heterozygote state with subtle clinical findings such as mild intellectual disability and modest creatine kinase elevation. In an additional family, a similarly affected male sibling died at 7 months of age due to severe muscle disease (3), while in another case the mother was not genetically tested but was reported to have low intellectual capacity (4). The described patient’s ophthalmologic phenotype including nystagmus, poor visual fixation, pale optic discs, and decreased caliber of the retinal vessels, was observed despite the deletion sparing the loci implicated in Xp21-associated retinal disease and is not emphasized in other reported and genotypically comparable cases. Deletions involving the Xp21 region, however extending centromerically of the region reported here, involve genes such as *RPGR* and *CACNA1F*, implicated in retinitis pigmentosa, Åland Island eye disease and congenital nystagmus (6–8). In a patient described by Behnecke et al., with an intragenic *IL1RAPL1* deletion, the reported ophthalmologic finding was strabismus (9). Additional cases are summarized in **Table 2**. Collectively, these observations suggest that this Xp21 deletion is frequently inherited from a mildly affected mother, with confirmed or suspected maternal transmission in four of the five cases. Heterozygous females having Xp21 deletions may be clinically unaffected (10) or may present with mild features, including mild to moderate intellectual disability, mild muscular involvement, or partial adrenal insufficiency. This variable expression is thought to be influenced by X-chromosome inactivation patterns (11, 12). These findings highlight the importance of considering maternal inheritance and suggest that X-chromosome inactivation studies in heterozygote females may provide valuable insights into clinical variability. The overall phenotype remains complex, reflecting both the extent of the deletion and the genes involved.

**Table 2:** A summary of the genetic, laboratory and clinical findings in the reported Xp21 deletion syndrome cases including the present one.

Patient	Age of clinical onset	Age at diagnosis of Xp21 deletion	Available molecular results	First symptoms	Later presentation	Cortisol	ACTH	CK	CK-MB	AST/ALT	(Pseudo)-Trigliceridemia in serum		(Glyceroluria mmol/mmol creatinine ref. 0.01-0.1)	Female carriers (signs)
Index case	First days	Neonatal	Deletion size: 4.5 Mb; [GRCh37]Xp21.3p21.2(26936592_31431584)x0	Muslce hypotonia, nystagmus intermittent diarrhea, regurgitation, slow weight gain, salt loss	Psychomotor delay, hypotonia, meningococcal sepsis and meningitis	13.9 µg/dL (ref. 7.78–13.90 µg/dL) (in short Synacthen test - 16µg/dL)	536 pg/mL (ref. 7.2–63.3 pg/mL)	3000 U/L (ref. 70–380 U/L)	211 U/L (ref. 7–25 U/L)	146/ 84 U/L	1149 mg/dL (ref. <150 mg/dL)	high		Mother (mild intellectual disability)
Manisha et al., 2026 (48)	DOL 2	Neonatal	Deletion size: 5.5 Mb involving 30 OMIM genes	Lethargy, poor feeding, frontal bossing, hyperpigmentation, cholestasis, hypotonia, salt loss	NR	5.4 µg/dL	>1250 pg/mL	2641.9 U/L	NR	NR	1580 mg/dL	high		NR
Stankovic et al., 2025 (49) 1^st^ patient	DOL 8	Neonatal dx. of PAI, dx. of DMD at 6 months of age	*DMD* deletion (only MLPA available)	Vomiting, poor feeding, weight loss, dehydration, skin hyperpigmentation, with salt loss	Hypotonia, he died at 7 months of age at home	1.3 µg/dL (ref. 15-50 µg/dL)	6870 pg/mL (ref. 7.2-66.3 pg/mL)	9516 U/L	NR	2316/547 U/L	629 mg/dL	NR		NR
Stankovic et al., 2025 (49) 2^nd^ patient	DOL 42	Infancy	Deletion size: 8.2 Mb; [hg19]Xp21.3-p21.1 (25119054_337723252)x0	Vomiting, poor feeding, weight loss, dehydration, skin hyperpigmentation	Psychomotor delay, diminished response to sounds, poor toy-handling skills, and reduced social interactions, with rare vocalizations and minimal social smile	2.2 µg/dL (ref. 15-50 µg/dL)	354 pg/mL	17750 U/L	NR	225/ 94 U/L	1045 mg/dL	NR		Mother (slightly raised CK)
Singin et al., 2025 (50)	Neonatal dx. of PAI	8 months	Deletion size: 8.6 Mb, [GRCh37]Xp21.3p21.1 (28514128_37189187)x0	Poor weight, microcephaly, hyperpigmentation, hypotonia, dysmorphy (upward-deviated eyes and low-set ears), cryptorchidism, adrenal crisis,	NR	0.8 ug/dl (neonatal) (ref. 4.3-22.4 µg/dL)	612 pg/mL (ref. 0–46 pg/mL (neonatal))	40800 U/L (ref. 46–171 U/L)		1081/293 (ref. 0-34/10–49 U/L)	637 mg/dL (ref. 0–150 mg/dL)	1465.14 mmol/mmol creatinine (normal range: 0.01-0.1)		Mother (mental disability, parents - first-degree consanguinity)
Korkut et al., 2016 (3) 1^st^ patient	DOL 36	2^nd^ month dx. of CGKD	Deletion including *NR0B1*, *GK*, a part of *DMD* and *IL1RAPL1*	Difficulty feeding, vomiting, weight loss, hyperpigmentation, hypotonia, dehydration, dysmorphic facial features	NR	12.6 µg/dL	>2000 pg/mL	5758 U/L (ref. 68–580 U/L)	NR	NR	1193 mg/dL (ref. 35–110 mg/dL)	4847.6 mmol/mmol creatinine ref. 0-40		NR
Korkut et al., 2016 (3) 2^nd^ patient	DOL 18	2^nd^ month dx. of CGKD	Deletion size: 3.88 Mb, including *GK*, *NR0B1*, *IL1RAPL1* and exon 45 extending through 3’ end of *DMD*)	Vomiting, and weight loss, dehydration, dysmorphic facial features	NR	20.6 µg/dL	628 pg/mL	28134U/L (ref. 68–580 U/L)	592 U/l (ref. 0–25 U/L	NR	761 mg/dL	NR		NR
Kalashnikova et al., 2025 (51)	DOL 1	Neonatal period - dx. of PAI; after 4 years - CGKD	Deletion size: 5.31 Mb; [GRCh38]Xp21.3p.21.1(28085320_33391678)×0	Hypoglycemia, elevated ACTH	Left-sided cryptorchidism, psychomotor developmental delay, pseudohypertrophy of the calves, muscular hypotonia	NR	DOL 1: ~2000 pg/mL at 2 years of age: 357 pg/mL (ref. ~23–209 pg/mL)	9661 U/L (ref. 0–190 U/L)	NR	240/299 U/L (ref. <40 U/L)	NR	NR		Mother
Wikiera et al., 2021 (52) 1^st^ patient	5 weeks	5 weeks dx. of PAI, at 1 year dx. of CGKD	Deletion of *NR0B1,GK and DMD* (only MLPA available)	Failure to thrive, loss of body weight, athrepsia, dehydration, weak muscle tone, respiratory tract infection, salt loss	Several infections of the respiratory and urinary tracts, delayed psychomotor development, severe undernutrition, at 8 years of age - calf hypertrophy, at 10 years of age - dilated cardiomyopathy, at 13 years of age - sepsis and death	1.7 µg/dL in short Synacthen test - 2.8 µg/dL	162 pg/mL (ref. <45 pg/mL)	13126 U/L (ref. < 154 U/l)	326–598 U/L, (ref. < 25 U/L)	NR	638 mg/dL	high		*De novo*
Wikiera at al. 2021 (52) 2^nd^ patient	5 weeks	5 weeks dx. of PAI, at 6 years dx. of CGKD	Deletion of *NR0B1,GK, IL1RAPL1* and C-terminal region of *DMD* (only MLPA available)	Dehydration, adynamia and failure to thrive, salt loss	Delayed psychomotor development, at 5 years of age: ischemic stroke, at 6 years of age: high CK and Tg, hypogonadotropic hypogonadism	low cortisol levels	elevated plasma ACTH level (NR)	4236 U/L (ref. <154 U/L)	NR	NR	590 mg/dL	NR		*De novo*
Sevim at al. 2011 (4)	1 month	2 to 3 months dx. of CGKD	Deletion size: 3.4 Mb; Xp21.2p21.3(27934242_31363324)x0	Dehydration, scrotal hyperpigmentation, hypotonia, salt loss	Delayed psychomotor development	1 month: 6.7 µg/dL (ref. 6.7-22.6 µg/dL) After ACTH stimulation test 66.5 µg/dL 11months: 4.7 µg/dL After ACTH stimutaion test: 11.0 µg/dL	20.8 pg/mL (ref. 0–227 pg/mL)	7019 U/L	NR	298.8/174 U/L	1275 mg/dL	high		Not performed in mother with intellectual disability
Gau et al., 2025 (2) 1^st^ patient	DOL 1	DOL 1 - dx. of PAI, early infancy dx. of CGKD	Deletion size: 2.4 Mb; [GRCh37]Xp21.1p21.3(29096607_31516964)x0 with partial gain at both ends (28611286_28829824)x3 and (31852489_32001787)x3	Hyperpigmentation, no discernible adrenal glands on abdominal US	Delayed psychomotor development	0.7 μg/dL (ref. 0.55-19.8 µg/dL)	6420 pg/mL (ref. 1.4-46.2 pg/mL)	22875 U/L (ref. 42–321 U/L)		265/313 U/L (ref. 25-68/13–55 U/L)	171 mg/dl (ref.<140)	high		Mother (high CK) and sister (high CK, motor delay, Gowers sign)
Fu et al., 2024 (23)	Infancy	2 years (chromosomal microarray)	Deletion size: 4.2 Mb; Xp21.3p21.1(29296579_33551038)x0	Global developmental delay, gross motor and speech delay, axial hypotonia, poor head control, inability to sit unassisted or walk	Dysmorphic features (absent eyebrows, temporal thinning, high forehead, frontal bossing), axial hypotonia with peripheral hypertonia, myopathy, PAI confirmed on ACTH-stimulation test	NR	156 pg/mL (ref. 6–48 pg/mL)	14809 U/L (ref. 27–160 U/L)	NR	307/265 U/L (ref. 20–60/15–45 U/L)	683 mg/dL (ref. 44–157 mg/dL)	Marked glyceroluria on GC–MS		Sister at 20 months of age (moderate hyperCKemia, moderate glyceroluria, mother (learning difficulties)
Bregvadze et al., 2025 (53) (elder sibling; younger sibling - same deletion, similar findings)	Neonatal	13 months; initially misdiagnosed as CAH	Deletion size: 6.6 Mb; [GRCh37]Xp21.3p21.1 (27478915_34150395)x0	At birth: hypotonia, hypospadias, scrotal hyperpigmentation, lethargy, vomiting, hyperkalemia, hyponatremia, salt loss	At 13 months of age: failure to thrive, axial hypotonia, dysmorphic facies (high forehead, frontal bossing, rounded palpebral fissures, long philtrum); sitting at 13 months, unable to stand or walk, intellectual delay, recurrent hypoglycemia 50–60 mg/dL	NR (low ACTH, aldosterone, DHEA-S)	4.24 pg/mL (ref. 4.7–48.8 pg/mL)	600 U/L (ref. <228 U/L)	NR	358/293 U/L (ref. 0–40/0–35 U/L	260 mg/dL (ref. <200 mg/dL)	NR		Mother (asymptomatic, confirmed carrier)
Sanz-Ruiz et al., 2009 (5)	7 months	Infancy stepwise dx. of DMD, GKD and of Xp21 deletion syndrome	Deletion size: 3 Mb; Xp21.3p22.1	Axial hypotonia, psychomotor and developmental delay, frontal bossing, strabismus, unilateral cryptorchidism, unstable sitting, no rolling, no salt loss, no neonatal adrenal crisis - unusual, predominantly myopathic presentation	At 3.5 years of age: intellectual disability, muscle weakness, no independent walking, intercurrent pneumonia and Salmonella bacteremia with ketotic hypoglycemia (glucose - 17 mg/dL). Brain MRI: delayed myelination	9.8 µg/dL (ref. 4-19.4 µg/dL)	>1,250 pg/mL (ref. 0–46 pg/mL)	12829 U/L (ref. <170 U/L)	NR	158/252 U/L (ref. 5–37/5–41 U/L)	544 mg/dL (ref. 50–200 mg/dL)	Urine glycerol 12,332 µM/mol creatinine (ref. 17–281);		Mother (carrier of the same deletion, mild CK elevation, maternal consanguinity, mild intellectual disability)

The original values in Kalashnikova et al. (440.4 pmol/L) and Sevim et al. (4.58 pmol/L) of ACTH concentrations were reported in pmol/L, for the sake of comparability with other values reported in the table the units were converted to pg/mL. Reference ranges given in brackets are reported as they were in the original studies referenced in the table. No reference value is reported if it was not included in the original study. NR, not reported; dx., diagnosis; CGKD, complex glycerol kinase deficiency; PAI, primary adrenal insufficiency; DMD, Duchenne muscular dystrophy; CAH, congenital adrenal hyperplasia; DOL, day of life; Tg, triglicerides.

The presented patient developed symptoms of PAI with SWS during the second week of life. The most common cause of PAI in neonates and infants is CAH with pathogenic variants in the *CYP21A2* gene encoding 21-hydroxylase, accounting for 90-95% of all cases. In the presented patient, PAI arose due to the involvement in the contiguous deletion of the *NR0B1* gene. The *NR0B1* encodes an orphan member of the nuclear receptor superfamily that functions as a transcriptional repressor and is expressed in steroidogenic and endocrine tissues, including adrenal cortex, pituitary gonadotroph cells, hypothalamus and testicles. Certain pathogenic variants, including deletions of *NR0B1*, cause X-linked congenital adrenal hypoplasia (AHC), often accompanied by hypogonadotropic hypogonadism and delayed puberty (13, 14). In the presented patient, the external genitalia were normal, and the same applied to FSH, LH and testosterone concentrations during the period of minipuberty (2^nd^ and 3^rd^ month of life). Copy number gains within the Xp21 region, that include *NR0B1*, may cause a dosage-sensitive gonadal dysgenesis (15, 16). In a cohort of 155 children with PAI of unexplained etiology presenting with SWS pathogenic variants in *NR0B1* were identified in 12 patients (7.7%). Patients usually presented as neonates, however, a more insidious onset of symptoms in later childhood is possible (14). Girls can be affected if skewed chromosome X inactivation occurs (12). Patients with AHC are at risk of an acute adrenal crisis. It can be precipitated by intercurrent illness, fever, vomiting, diarrhea, trauma, or seizures, and manifests with hypotension, shock, hypoglycemia, hyponatremia, and hyperkalemia (14). In Xp21 deletion syndrome, the co-existence of GKD might complicate its clinical course.

Another contiguously deleted gene identified in the child was the *GK* gene. *GK* encodes glycerol kinase, which catalyzes the phosphorylation of glycerol to glycerol-3-phosphate, a key intermediate of gluconeogenesis. GKD may be asymptomatic particularly in adults or associated with severe metabolic disturbances in children. Reported clinical manifestations of isolated GKD include ketoacidosis, fever, gastrointestinal symptoms, episodic hypoglycemia, hypothermia, apnea, and altered mental status. GKD is characterized by glyceroluria and elevated plasma glycerol concentration as high as 8 mmol/l, while normally it does not exceed 0.28 mmol/l. Urinary glycerol excretion may exceed 150 mmol/L, while in healthy individuals it is negligible (17, 18). There is no correlation between the genotype and phenotype in isolated GKD, that would allow predicting which patients are at increased risk of episodic hypoglycemia (18). However, in Xp21 deletion syndrome, the risk might be increased by *NR0B1*-related AHC and consequent glucocorticoid deficiency. The elevated urinary glycerol excretion may also contribute to osmotic dehydration during episodes of increased catabolism (19). Elevated serum glycerol concentration causes pseudo-hypertriglyceridemia, as routine enzymatic assays measure total glycerol, including all acylglycerol species, rather than true triglyceride concentration, which is a potential diagnostic pitfall (20, 21). This underscores the importance of interpreting lipid profiles in clinical context and confirms the utility of GC-MS and LC-MS/MS in evaluating suspected IMD (21–23). Clinical management of patients with isolated GKD consists primarily of frequent follow-up visits, in selected cases a fat-restricted diet with avoidance of prolonged fasting (18). In cases of isolated GKD presenting with acute metabolic decompensation, appropriate management involves treating the condition as a fasting-intolerance emergency, thereby to maintain euglycemia, suppress ketosis and catabolism e.g. by intravenous glucose and a multi-electrolyte solution. However, no large systematic studies determining the optimal treatment protocol for decompensated GKD have been published to date.

The deletion identified in the proband encompassed 266–477 bp within the distal region of the *DMD* gene (NM_004006.3, exons 61 to 79). *DMD* encodes dystrophin and is the largest known human gene. It is associated with Duchenne (DMD) and Becker (BMD) muscular dystrophies. In muscle fibers, dystrophin is a part of a chain of proteins linking the actin cytoskeleton and the basal lamina (24). One hallmark of dystrophinopathies is an elevation of CK, often reaching levels 50 to 100 times the upper limit. CK concentrations are typically highest early in the disease course and may decline as the disease progresses (25). In addition to hyperCKemia, the described patient also exhibited elevated levels of CK-MB, NT-proBNP, and troponin T. However, cardiac studies (ECHO and ECG) revealed no evidence of significant abnormalities, including cardiomyopathy, which in patients with DMD/BMD becomes invariably evident by late teens (26). The direct causes of cardiac dysfunction in DMD/BMD patients remain elusive, however increased fibrosis, disrupted calcium homeostasis, accelerated senescence, intercalated disc disarray and disrupted membrane integrity have been demonstrated in *in-vitro* and animal models (27–30). Lamotrigine exposure during pregnancy was initially considered a potential contributor to the observed cardiac laboratory abnormalities and elevated concentrations of transaminases. However, the absence of detectable lamotrigine in the patient’s blood, no apparent symptoms of lamotrigine withdrawal, and the confirmed genetic diagnosis argue against drug-related symptoms and toxicity. The significant elevation of cardiac parameters and liver function tests in the presented case were likely secondary to the combined impact of the multi-gene deletion on the myocardium and skeletal muscle. In patients with DMD/BMD elevated liver function tests have been reported and the transaminases’ serum activity has been traced back to skeletal muscle, not only the liver. The same applies to the cardiac markers, being partly released from the skeletal muscle (25). Lamotrigine is generally regarded as relatively safe during pregnancy, with the majority of neonatal adverse effects being mild and transient, including sleep disturbances, gastrointestinal symptoms, or hypotonia with poor feeding (31). However, fetal cardiac anomalies in children born to mothers taking lamotrigine while pregnant have been reported (32). Additional case-relevant adverse effects of lamotrigine were myocarditis in a 14-year-old patient (33), hepatitis (34), and myotoxicity with rhabdomyolysis, albeit after intentional overdose (35), which is why it was at initial presentation considered a possible contributor to the patients clinical picture, but was excluded as such. Nevertheless, cardiac involvement is a major cause of morbidity and mortality in patients with dystrophinopathies. Proactive, specialist cardiologic surveillance is strongly recommended for DMD patients, and should analogously be extended to those with Xp21 deletion syndrome (36–39).

Another gene that was affected by the deletion in the described patient and will likely affect his further development is *IL1RAPL1*. *IL1RAPL1* is expressed in brain regions involved in engram formation. The protein it encodes for is a member of the interleukin 1 receptor family and plays a role in synapse formation and stabilization (40, 41). Long-term follow-up of the patient and his family is crucial to adequately assess cognitive development, given his increased risk of intellectual disability. This risk is attributable not only to the *IL1RAPL1* deletion (40), but also to the partial *DMD* deletion, which is predicted to affect *DMD* gene products, implicated in cognitive impairment in DMD/BMD patients (42, 43). Although significant structural ocular abnormalities and primary retinal gene defects were not identified in this patient, the contiguous gene deletion encompassing *IL1RAPL1*, and potentially additional neuronal genes in the Xp21.3-p21.2 region, supports a central visual processing deficit (40). Individuals with DMD/BMD may present with proliferative retinopathy, cataracts, electroretinographic abnormalities, impaired contrast sensitivity, color vision loss, and elevated flash detection thresholds during dark adaptation. It is thought to be caused by the absence of dystrophin isoforms normally transcribed from internal promoters in the retina and crystalline lens (43). We speculate that the hemizygous deletion of the Xp21.3-p21.2 region, encompassing *IL1RAPL1* and partially *DMD*, may contribute to the patient’s profound visual impairment and developmental delay. Loss of *IL1RAPL1* could potentially interfere with synapse formation and neuronal connectivity, thereby affecting central visual processing (40, 41). Additionally, the deletion involved *DMD* regions, from which transcripts for dystrophin isoforms like Dp71 and Dp40 are derived (see **Figure 1**). The absence of those has been shown to affect the central nervous system and influence motor development in DMD/BMD patients, which could further exacerbate neuronal and sensory dysfunction (42, 44–47).

In conclusion, we report a patient with the ultra-rare Xp21 deletion syndrome, and illustrate the importance of early molecular diagnosis, which enabled timely hormone replacement, metabolic surveillance, cardiologic follow-up, and structured neurodevelopmental care.

## Data Availability

The original contributions presented in the study are included in the article/supplementary material. Further inquiries can be directed to the corresponding author.

## References

[1] Pizza A Picillo E Onore ME Scutifero M Passamano L Nigro V . Xp21 contiguous gene deletion syndrome presenting as Duchenne muscular dystrophy and glycerol kinase deficiency associated with intellectual disability: case report and review literature. Acta Myol. (2023) 42:24–30. doi: 10.36185/2532-1900-246 37091526 PMC10115399

[2] Gau M Iemura R Orimoto R Adachi E Saito Y Yamano H . Congenital adrenal hypoplasia with neurodevelopmental delay due to contiguous Xp21 deletion: a case series with review of literature. Endocr J. (2026) 73:483–93. doi: 10.1507/endocrj.EJ25-0265 41285479 PMC12996724

[3] Korkut S Baştuğ O Raygada M Hatipoğlu N Kurtoğlu S Kendirci M . Complex glycerol kinase deficiency and adrenocortical insufficiency in two neonates. J Clin Res Pediatr Endocrinol. (2016) 8:468–71. doi: 10.4274/jcrpe.2539 27087023 PMC5198007

[4] Sevim U Fatma D Ihsan E Gulay C Nevin B . A neonate with contiguous deletion syndrome in XP21. J Pediatr Endocrinol Metab. (2011) 24:1095–8. doi: 10.1515/jpem.2011.350 22308874

[5] Sanz-Ruiz I Bretón-Martínez JR Del Castillo-Villaescusa C Cásanovas-Martínez A Martínez-Castellano F Millán-Salvador JM . Contiguous gene deletion syndrome in Xp21: an unusual form of presentation. Rev Neurol. (2009) 49:472–4 19859888

[6] Duemler A Gao H Powell J Iannaccone A Alekseev O . land Island eye disease in two patients harboring novel CACNA1F variants. Ophthalmic Genet. (2025) 46:495–8. doi: 10.1080/13816810.2025.2505914 40400241 PMC12353906

[7] Lin S Vermeirsch S Pontikos N Martin-Gutierrez MP Daich Varela M Malka S . Spectrum of genetic variants in the most common genes causing inherited retinal disease in a large molecularly characterized United Kingdom cohort. Ophthalmol Retina. (2024) 8:699–709. doi: 10.1016/j.oret.2024.01.012 38219857 PMC11932969

[8] Dumitrescu AV Pfeifer WL Arhens M Andorf JL Drack AV . CACNA1F-related synaptic dysfunction: challenges diagnosing congenital stationary night blindness presenting without night blindness. Can J Ophthalmol. (2024) 59:e808–18. doi: 10.1016/j.jcjo.2023.11.022 38159912

[9] Behnecke A Hinderhofer K Bartsch O Nümann A Ipach ML Damatova N . Intragenic deletions of IL1RAPL1: Report of two cases and review of the literature. Am J Med Genet Part A. (2011) 155:372–9. doi: 10.1002/ajmg.a.33656 21271657

[10] Zhang LJ Liu WL Shao SY Xu Y Zhou L . Genetic analysis of an asymptomatic female with a large Xp deletion revealed insights into the X chromosome inactivation pattern: a case report. Mol Cytogenet. (2025) 18:24. doi: 10.1186/s13039-025-00729-0 41029434 PMC12487153

[11] Heide S Afenjar A Edery P Sanlaville D Keren B Rouen A . Xp21 deletion in female patients with intellectual disability: Two new cases and a review of the literature. Eur J Med Genet. (2015) 58:341–5. doi: 10.1016/j.ejmg.2015.04.003 25917374

[12] Shaikh MG Boyes L Kingston H Collins R Besley GTN Padmakumar B . Skewed X inactivation is associated with phenotype in a female with adrenal hypoplasia congenita. J Med Genet. (2008) 45:e1. doi: 10.1136/jmg.2007.055129 18762570 PMC2602739

[13] Zhang W Li Y Chen S Zhang C Chen L Peng G . nr0b1 (DAX1) loss of function in zebrafish causes hypothalamic defects via abnormal progenitor proliferation and differentiation. J Genet Genomics. (2022) 49:217–29. doi: 10.1016/j.jgg.2021.08.019 34606992

[14] Buonocore F Maharaj A Qamar Y Koehler K Suntharalingham JP Chan LF . Genetic analysis of pediatric primary adrenal insufficiency of unknown etiology: 25 years’ Experience in the UK. J Endocr Soc. (2021) 5:bvab086. doi: 10.1210/jendso/bvab086 34258490 PMC8266051

[15] García-Acero M Molina M Moreno O Ramirez A Forero C Céspedes C . Gene dosage of DAX-1, determining in sexual differentiation: duplication of DAX-1 in two sisters with gonadal dysgenesis. Mol Biol Rep. (2019) 46:2971–8. doi: 10.1007/s11033-019-04758-y 30879272

[16] Zheng XQ Zhou QL Gu W . 46,XY disorders of sex development and muscular dystrophy caused by Xp21 duplication: a case report and literature review. Transl Pediatr. (2024) 13:2088–96. doi: 10.21037/tp-24-327 39649651 PMC11621888

[17] Shah A Xu H Kwon HJ Wondisford FE . *In vivo* glycerol metabolism in patients with glycerol kinase deficiency. JIMD Rep. (2024) 65:392–400. doi: 10.1002/jmd2.12452 39512433 PMC11540572

[18] Lamiquiz-Moneo I Mateo-Gallego R Fernández-Pardo J López-Ariño C Marco-Benedí V Bea AM . Glycerol kinase deficiency in adults: Description of 4 novel cases, systematic review and development of a clinical diagnostic score. Atherosclerosis. (2020) 315:24–32. doi: 10.1016/j.atherosclerosis.2020.10.897 33212314

[19] Wu JW Yang H Wang SP Soni KG Brunel-Guitton C Mitchell GA . Inborn errors of cytoplasmic triglyceride metabolism. J Inherit Metab Dis. (2015) 38:85–98. doi: 10.1007/s10545-014-9767-7 25300978

[20] Backes JM Dayspring TD Hoefner DM Contois JH McConnell JP Moriarty PM . Identifying pseudohypertriglyceridemia in clinical practice. Clin Lipidology. (2014) 9:625–41. doi: 10.2217/clp.14.52

[21] Pant V Pyakurel D Gautam K Pradhan S . Pseudo-hypertriglyceridaemia in glycerol kinase deficiency misdiagnosed and treated as true hypertriglyceridaemia. BMJ Case Rep. (2022) 15:e248251. doi: 10.1136/bcr-2021-248251 35292548 PMC8928288

[22] Sriphrapradang C Srisawasdi P Shantavasinkul PC Auparakkitanon S Krongvorakul J Punprasit S . Falsely elevated triglyceride and lipase levels due to hyperglycerolemia in a burn patient treated with topical silver sulfadiazine. J Clin Lipidology. (2024) 19(1):162–6. doi: 10.1016/j.jacl.2024.10.006 39586762

[23] Fu X Williamson CP Bosfield K . Pseudo-hypertriglyceridemia in a 2-year-old male with global developmental delay, myopathy and adrenal hypoplasia. J Mass Spectrom Adv Clin Lab. (2024) 32:47–9. doi: 10.1016/j.jmsacl.2024.02.004 38419979 PMC10900099

[24] Liu S Su T Xia X Zhou ZH . Native DGC structure rationalizes muscular dystrophy-causing mutations. Nature. (2024) 637:1261–71. doi: 10.1038/s41586-024-08324-w 39663457 PMC11936492

[25] Rohlenová M Machová K Baranová J Mokrá L Mensová L Mazanec R . Serum creatine kinase and transaminase levels in duchenne and becker muscular dystrophies. Muscle Nerve. (2025) 72:240–9. doi: 10.1002/mus.28431 40350776

[26] Broomfield J Abrams K Latimer N Guglieri M Rutherford M Crowther M . Natural history of Duchenne muscular dystrophy in the United Kingdom: A descriptive study using the Clinical Practice Research Datalink. Brain Behav. (2023) 13:e3331. doi: 10.1002/brb3.3331 37957895 PMC10726817

[27] Souidi M Resta J Dridi H Sleiman Y Reiken S Formoso K . Ryanodine receptor dysfunction causes senescence and fibrosis in Duchenne dilated cardiomyopathy. J Cachexia Sarcopenia Muscle. (2024) 15:536–51. doi: 10.1002/jcsm.13411 38221511 PMC10995256

[28] Zhou Z Xu R Cai X Fu H Xu K Yuan W . Association between myocardial oxygenation and fibrosis in duchenne muscular dystrophy: analysis by rest oxygenation-sensitive magnetic resonance imaging. J Magn Reson Imaging. (2024) 60:1989–99. doi: 10.1002/jmri.29273 38328865

[29] Taglietti V Kefi K Mirciloglu B Bastu S Masson JD Bronisz-Budzyńska I . Progressive cardiomyopathy with intercalated disc disorganization in a rat model of Becker dystrophy. EMBO Rep. (2024) 25:4898–920. doi: 10.1038/s44319-024-00249-9 39358550 PMC11549483

[30] Houang EM Sham YY Bates FS Metzger JM . Muscle membrane integrity in Duchenne muscular dystrophy: recent advances in copolymer-based muscle membrane stabilizers. Skeletal Muscle. (2018) 8:31. doi: 10.1186/s13395-018-0177-7 30305165 PMC6180502

[31] Cohen‐Israel M Berger I Martonovich EY Klinger G Stahl B Linder N . Short‐ and long‐term complications of in utero exposure to lamotrigine. Brit J Clin Pharma. (2018) 84:189–94. doi: 10.1111/bcp.13437 29044597 PMC5736833

[32] Morrow J Russell A Guthrie E Parsons L Robertson I Waddell R . Malformation risks of antiepileptic drugs in pregnancy: a prospective study from the UK Epilepsy and Pregnancy Register. J Neurol Neurosurg Psychiatry. (2006) 77:193–8. doi: 10.1136/jnnp.2005.074203 16157661 PMC2077578

[33] Bayhan T Şahin M Yıldırım I Karagöz T . Lamotrigine related myocarditis: case report. Arch Turkish Soc Cardiol. (2012) 40:358–60. doi: 10.5543/tkda.2012.70268 22951854

[34] Moeller KE Wei L Jewell AD Carver LA . Acute hepatotoxicity associated with lamotrigine. AJP. (2008) 165:539–40. doi: 10.1176/appi.ajp.2007.07050728 18381923

[35] Siniscalchi A Mintzer S De Sarro G Gallelli L . Myotoxicity Induced by Antiepileptic Drugs: Could be a Rare but Serious Adverse Event? Psychopharmacol Bull. (2021) 51:105–16. doi: 10.64719/pb.4421 34887602 PMC8601760

[36] Villa C Auerbach SR Bansal N Birnbaum BF Conway J Esteso P . Current practices in treating cardiomyopathy and heart failure in duchenne muscular dystrophy (DMD): understanding care practices in order to optimize DMD heart failure through ACTION. Pediatr Cardiol. (2022) 43:977–85. doi: 10.1007/s00246-021-02807-7 35024902 PMC8756173

[37] Hakimi M Burnham T Ramsay J Cheung JW Goyal NA Jefferies JL . Electrophysiologic and cardiovascular manifestations of Duchenne and Becker muscular dystrophies. Heart Rhythm. (2025) 22:192–202. doi: 10.1016/j.hrthm.2024.07.008 38997055

[38] Landfeldt E Alemán A Abner S Zhang R Werner C Tomazos I . Predictors of cardiac disease in duchenne muscular dystrophy: a systematic review and evidence grading. Orphanet J Rare Dis. (2024) 19:359. doi: 10.1186/s13023-024-03372-x 39342355 PMC11439250

[39] Esteso P Auerbach SR Bansal N Harris R Soslow JH Birnbaum BF . Cardiac treatment for Duchenne muscular dystrophy: consensus recommendations from the ACTION muscular dystrophy committee. Cardiol Young. (2025) 35:770–5. doi: 10.1017/S1047951125000587 40012319

[40] Montani C Gritti L Beretta S Verpelli C Sala C . The synaptic and neuronal functions of the X-linked intellectual disability protein interleukin-1 receptor accessory protein like 1 (IL1RAPL1). Dev Neurobiol. (2019) 79:85–95. doi: 10.1002/dneu.22657 30548231

[41] Valnegri P Montrasio C Brambilla D Ko J Passafaro M Sala C . The X-linked intellectual disability protein IL1RAPL1 regulates excitatory synapse formation by binding PTPδ and RhoGAP2. Hum Mol Genet. (2011) 20:4797–809. doi: 10.1093/hmg/ddr418 21926414 PMC3221541

[42] Naidoo M Anthony K . Dystrophin dp71 and the neuropathophysiology of duchenne muscular dystrophy. Mol Neurobiol. (2020) 57:1748–67. doi: 10.1007/s12035-019-01845-w 31836945 PMC7060961

[43] Barboni MTS Joachimsthaler A Roux MJ Nagy ZZ Ventura DF Rendon A . Retinal dystrophins and the retinopathy of Duchenne muscular dystrophy. Prog Retin Eye Res. (2023) 95:101137. doi: 10.1016/j.preteyeres.2022.101137 36404230

[44] García-Cruz C Aragón J Lourdel S Annan A Roger JE Montanez C . Tissue- and cell-specific whole-transcriptome meta-analysis from brain and retina reveals differential expression of dystrophin complexes and new dystrophin spliced isoforms. Hum Mol Genet. (2023) 32:659–76. doi: 10.1093/hmg/ddac236 36130212 PMC9896479

[45] Catapano F Alkharji R Chambers D Singh S Aghaeipour A Malhotra J . A comprehensive spatiotemporal map of dystrophin isoform expression in the developing and adult human brain. Acta Neuropathol Commun. (2025) 13:110. doi: 10.1186/s40478-025-01996-z 40400011 PMC12096690

[46] Chesshyre M Ridout D Stimpson G Ricotti V De Lucia S Niks EH . Dystrophin isoform deficiency and upper-limb and respiratory function in Duchenne muscular dystrophy. Dev Med Child Neurol. (2025) 67:1280–9. doi: 10.1111/dmcn.16282 40084496 PMC12426305

[47] Chesshyre M Ridout D Stimpson G Servais L Baranello G Manzur A . Influence of dystrophin isoform deficiency on motor development in duchenne muscular dystrophy. Ann Clin Transl Neurol. (2025) 12:1732–42. doi: 10.1002/acn3.70097 40552660 PMC12455871

[48] Manisha P K K S Thomas D . Complex glycerol kinase deficiency: A case of segmental loss of xp chromosome. Indian J Pediatr. (2026) 93:671–. doi: 10.1007/s12098-026-06148-2 42012606

[49] Stankovic S Stankovic T Milica I Jakovljevic M Andrejevic M . The Severe neonatal presentation of Xp21 contiguous gene deletion: adrenal crisis and neuromuscular involvement. Acta Myologica. (2025) 44(3):109–11. doi: 10.36185/2532-1900-1417 41199733 PMC12599579

[50] Singin B Donbaloğlu Z Barsal Çetiner E Bedel A Çetin K Akcan Paksoy B . Xp21 contiguous gene deletion syndrome: diagnosis, treatment, and a review of the literature on a rare genetic disorder. J Clin Res Pediatr Endocrinol. (2025) 18(Suppl 1):83–91. doi: 10.4274/jcrpe.galenos.2025.2024-12-4 40103355 PMC13197103

[51] Kalashnikova TP Malov AG Veselkova AV Silaev AA . Xp21 contiguous gene deletion syndrome. Neurosci Behav Physi. (2025) 55:1179–82. doi: 10.1007/s11055-025-01875-z 40350738

[52] Wikiera B Jakubiak A Łaczmanska I Noczyńska A Śmigiel R . Complex glycerol kinase deficiency - long-term follow-up of two patients. Pediatr Endocrinol Diabetes Metab. (2021) 27:227–31. doi: 10.5114/pedm.2021.109681 34743506 PMC10228193

[53] Bregvadze K Kheladze N Tatishvili NN Dikhaminjia N Ghughunishvili M Tchankvetadze S . Genetic and clinical characterization of complex glycerol kinase deficiency in two male siblings: a case report. Clin Med Insights Endocrinol Diabetes. (2025) 18:11795514251317419. doi: 10.1177/11795514251317419 40171039 PMC11960146

